# Spray in Antiseptic Surgery

**Published:** 1880-06

**Authors:** 


Spray in Antiseptic Surgery.—In an article on antiseptic surgery, by Professor Lister, in the February number of the *Medical News and Abstract*, he again reminds the profession, that "if the spray is to be effectual, to be particularly careful to have a thoroughly reliable apparatus for its production."

It is well known to those acquainted with antiseptic surgery, that the ordinary apparatus is unreliable and sometimes unsafe; that boilers have exploded; lamps have gone out and the spray stopped in various ways, just at that critical moment.

Having seen these embarrassments, while a student in Edinburgh, some years since, I then and there suggested a method by which these objections could be overcome, in those buildings which are heated by steam. It is so simple and inexpensive that it can be used in any hospital where steam is used for heating, or where it is not, a bellows can be attached which will answer every purpose. An atomizer of this kind is now used in the University hospital.

One arm of a long atomizing tube is thrust through the cork of a quart bottle (the ordinary acetate of potash bottle), and the other is attached to a steam radiator or bellows by a rubber tube. The pressure is regulated by a stop-cock on the horizontal arm.

The one first used was made of glass tubes supported by a tin shoulder, and cost twenty-five cents. The pressure was regulated by a stop-cock at the radiator.

When the *expense*, convenience and safety of this simple atomizer are compared with those now in use its advantages will be apparent.

